# Interplay of defect levels and rare earth emission centers in multimode luminescent phosphors

**DOI:** 10.1038/s41467-022-35366-3

**Published:** 2022-12-08

**Authors:** Xinquan Zhou, Lixin Ning, Jianwei Qiao, Yifei Zhao, Puxian Xiong, Zhiguo Xia

**Affiliations:** 1 State Key Laboratory of Luminescent Materials and Devices, Guangdong Provincial Key Laboratory of Fiber Laser Materials and Applied Techniques, Guangdong Engineering Technology Research and Development Center of Special Optical Fiber Materials and Devices, School of Materials Science and Engineering, South China University of Technology, 510641 Guangzhou, China; 2 Anhui Key Laboratory of Optoelectric Materials Science and Technology, Key Laboratory of Functional Molecular Solids, Ministry of Education, Anhui Normal University, 241000 Wuhu, China; 3 School of Physics and Optoelectronics, South China University of Technology, 510641 Guangzhou, China

**Keywords:** Optical materials and structures, Materials for optics

## Abstract

Multimode luminescence generally involves tunable photon emissions in response to various excitation or stimuli channels, which demonstrates high coding capacity and confidentiality abilities for anti-counterfeiting and encryption technologies. Integrating multimode luminescence into a single stable material is a promising strategy but remains a challenge. Here, we realize distinct long persistent luminescence, short-lived down/upconversion emissions in NaGdTi_2_O_6_:Pr^3+^, Er^3+^ phosphor by emloying interplay of defect levels and rare earth emission centers. The materials show intense colorful luminescence statically and dynamically, which responds to a wide spectrum ranging from X-ray to sunlight, thermal disturbance, and mechanical force, further allowing the emission colors manipulable in space and time dimensions. Experimental and theoretical approaches reveal that the Pr^3+^ ↔ Pr^4+^ valence change, oxygen vacancies and anti-site Ti_Gd_ defects in this disordered structure contributes to the multimode luminescence. We present a facile and nondestructive demo whose emission color and fade intensity can be controlled via external manipulation, indicating promise in high-capacity information encryption applications.

## Abstract (web summary)

Information encryption technology calls for versatile multi-mode luminescent materials. Here, the authors develop phosphors with five integrated luminescence modes by exploiting the interplay of defect levels and rare-earth emission centers.

## Introduction

Counterfeiting phenomena are widely present in all aspects of economic and social life, and become a global security threat to both individuals and society^1^. Accordingly, advanced anti­counterfeiting strategies and technologies are in high demand nowadays, and thus the luminescence anti­counterfeiting has received growing attention due to the attractive properties of luminescent materials used, such as colorful and visually identifiable emissions, diverse emission lifetimes, and distinct emission modes in terms of different excitation channels.

Generally, the emission color and intensity provide spatial fingerprints, while the emission lifetime can offer an additional time dimension to temporally regulate luminescence that is advantageous to avoid emission overlap and background interference^2,3^. Normally, the emission modes include down- and up-converted photoluminescence (DCL and UCL), thermo- and photo-stimulated luminescence (TSL and PSL), X-ray excited optical luminescence (XEOL)^4,5^ and mechano-luminescence (ML), which was first implemented in information safety by Dai Nippon Printing in 2014^6^.

In the past decades, many types of luminescent materials have been explored for anti­counterfeiting, such as carbon dots^2,7^, nanocrystals^8^, metal halide perovskites^9–11^, transparent glass medium^12,13^, organic polymers^14,15^, and lanthanide/transition metal ions-doped inorganic phosphors^16–19^. Generally, multimode luminescence is realized by mixing materials with respective characteristic emissions that can be excited by different external stimuli sources. Nevertheless, such composite materials usually suffer from low luminescence efficiency due to performance mismatch and physicochemical incompatibility^20–22^. Moreover, integrating multimode luminescence into a single stable material is a promising strategy to achieve multifunctional applications, which still remains a challenge for next-generation luminescence anti-counterfeiting technology.

To develop multimode luminescence in a single material, trivalent lanthanide (Ln^3+^)-activated inorganic phosphors have attracted significant attention owing to their unique properties such as abundant 4*f* ^*N*^ energy levels, characteristic sharp line emissions, and the high chemical stability of inorganic hosts^23^. To be frank, this is not an easy task considering the complex interactions between multiple energy levels of rare earth and intrinsic defect levels. For example, for TSL and ML, the dopants should have multiple stable valence states (e.g., Pr^3+^ and Pr^4+^) and the host should contain defect levels with appropriate trap depths to capture (and release) charge carriers during (and after) the excitation. The trapping centers can be intrinsic and/or extrinsic defects, which play a vital role in realizing the temporally regulated luminescence. However, the nature of such defects and the basic insights into the trapping and de-trapping processes are largely elusive, which could hinder the continued development of dynamic luminescence anti­counterfeiting in the time dimension.

**Fig. 1:**
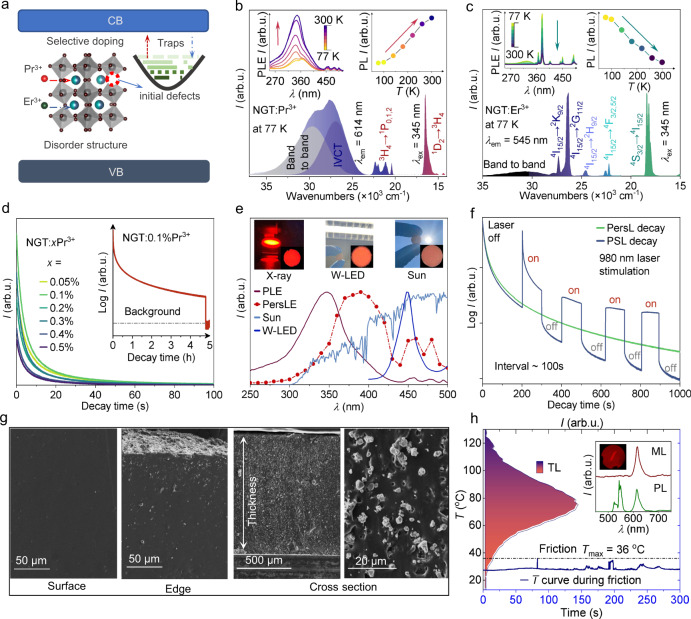
Multimode luminescence of Pr^3+^ and/or Er^3^^+^-doped NGT phosphors. **a** Sequential process leading to multimode luminescence via selective doping Pr^3+^ and Er^3+^ ions into the disordered NGT. CB, conduction band; VB, valence band. **b** Photoluminescence (PL) and PL excitation (PLE) spectra of NGT:0.1%Pr^3+^ at 77 K. The excitation spectrum is fitted by Gaussian functions. Insets are the temperature dependent PLE spectra and PL intensities. *I*, intensity; arb. u., arbitrary units; *λ*, wavelength; *T*, temperature; ex, excitation; em, emission. **c** PLE and PL spectra of NGT:2%Er^3+^ at 77 K. Insets show the temperature dependent PLE spectra and PL intensities. **d** Persistent luminescence (PersL) decay curves of NGT:*x*Pr^3+^ (*x* = 0.05%–0.5%) monitored at 614 nm after 365 nm excitation for 1 min. Inset shows the PersL decay curve of NGT:0.1%Pr^3+^ recorded for 5 h. **e** Comparison of solar spectrum, white light emitting diode (W-LED, bule chip + Y_3_Al_5_O_12_:Ce^3+^) spectrum, excitation spectrum of PersL (PersLE), and PLE of NGT:0.1%Pr^3+^ at room temperature (RT). Insets are the PL and PersL photographs under/after different light sources irradiation (the larger sizes photographs showing in Supplementary Fig. 1e). **f** PersL and photo-stimulated luminescence (PSL) decay curves of the NGT:0.1%Pr^3+^ monitored at 614 nm after 365 nm excitation for 1 min. During the PersL decays in a natural way, 0.5 W 980 nm continuous wave (CW) laser was introduced at an interval of 100 s to stimulate the sample for 100 s. **g** SEM images of surface, edge and cross-section (thickness of ca. 1 mm) of the flexible composite film consisting of NGT:0.1%Pr^3+^, 0.3%Er^3+^ particles embedded in the PDMS matrix. **h** Thermoluminescence (TL) spectrum of NGT:0.1%Pr^3+^, 0.3%Er^3+^ and the temperature curve under repeated frictions of the film. Inset shows the comparison of tribo-mechano-luminescence (ML) and PL spectra and photographs of the flexible composite film (the larger sizes photographs showing in Supplementary Fig. 1f). *T*_max_, maximum of temperature. Source data are provided as a Source Data file.

In this study, we synergistically integrated five-mode luminescence (DCL, UCL, TSL, PSL, and ML) by multiplexing the distinct emissions of Pr^3+^ and Er^3+^ in the NaGdTi_2_O_6_ (NGT) host. Herein, NGT crystallizes in the orthorhombic symmetry and the Na^+^ and Gd^3+^ occupy the same site with the occupancy ratio 1:1^24^. The Na/Gd site occupation disorder is beneficial to creating intrinsic defects in the host crystal, making it possible for the construction of suitable charge carrier-trapping centers (Fig. 1a). NGT:Pr^3+^, Er^3+^ phosphor shows intense colorful luminescence statically and dynamically, in response to various external stimuli (X-ray to near-infrared (NIR) light, thermal disturbance, and mechanical force). The main intrinsic defects are revealed by first-principles calculations, based on which the mechanistic origins of the dynamical luminescence processes are elucidated. A multilevel luminescence anti­counterfeiting scheme was designed, demonstrating capabilities of multichannel encoding, time-resolution display, and multi-stimulus-responsive decryption of visual information in daylight and dark environments. This work shows that the target combination of lanthanide dopants and inorganic hosts is a rational approach to advanced luminescence anti-counterfeiting.

## Results

### Microstructures and multimode luminescence behaviors

X-ray diffraction patterns of the undoped and doped samples are compared with the standard pattern of NGT in Supplementary Fig. 1a and no impurity phase was detected. Moreover, the ionic radii of Pr^3+^ and Er^3+^ ions are close to that of Gd^3+^ in the same fold of coordination, and thus the two dopants are expected to be located at Na/Gd sites in NGT. Supplementary Fig. 1b shows the scanning electron microscope images (SEM) and energy-dispersive X-ray spectroscopy patterns of the NGT:2%Pr^3+^, 2%Er^3+^ sample. The sample demonstrates high crystallinity and shapes into cubic-like individual microparticles with the particle size less than 10 µm. Obvious signals of all elements could be detected and these elements distribute uniformly over the particle, which further supports the successful incorporation of Pr^3+^, Er^3+^ ions in this structure.

Photoluminescence excitation (PLE) and photoluminescence (PL) spectra of Pr^3+^- and Er^3+^-singly doped samples at 77 K are displayed in Fig. 1b, c, respectively. By monitoring the Pr^3+ 1^D_2_ → ^3^H_4_ red emission at 614 nm, NGT:0.1%Pr^3+^ displays an intense broad PLE band in the range of 280–420 nm and several much weaker peaks in the range of 440–500 nm. The former broad band is attributed to a superposition of the host excitonic absorption and the intervalence charge transfer (IVCT, Pr^3+^/Ti^4+^–Pr^4+^/Ti^3+^) absorption on the longer-wavelength side. Gaussian deconvolution of this band revealed that the maximum host excitonic absorption is located at 29523 cm^–1^ (Fig. 1b), from which the host band gap is estimated to be 3.81 eV after taking into account the electron-hole binding energy of the exciton^25^. The latter absorption peaks in the blue spectral region are assigned to ^3^H_4_ → ^3^P_J_ transitions of Pr^3+^. With increasing temperature from 77 to 300 K, the intensities of all the absorption bands were enhanced. Specifically, the intensity of the host excitonic absorption increased more rapidly than that of the IVCT absorption, leading to a blue-shift of the absorption maximum (the upper left of Fig. 1b). This means that the host sensitization of the Pr^3+^ red emission is more efficient than the IVCT at higher temperature, owing to the thermally activated exciton hopping migration^26^. For NGT:2%Er^3+^, the PLE spectra of the Er^3+ 4^S_3/2_ → ^4^I_15/2_ green emission consists of a series of sharp lines attributed to the 4 *f* → 4 *f* transitions of Er^3+^ (as designated in Fig. 1c). The temperature dependence of these PLE spectra as well as the PL spectra (the top panel of Fig. 1c) shows expected behaviors, i.e., the intensities diminish with increasing temperature due to thermal quenching effect.

For Pr^3+^ singly doped samples, red persistent luminescence (PersL i.e., TSL at room temperature (RT)) was observed after switching off the excitation. Fig. 1d depicts the PersL decay curves of NGT:*x*Pr^3+^ (*x* = 0.05–0.5%) after ceasing the ultraviolet (UV) excitation at 365 nm. It shows that the optimal concentration is at *x* = 0.1%, for which the red luminescence persists for more than 5 h after being charged for 5 min (inset of Fig. 1d). Further increase of Pr^3+^ concentration results in a reduction of the PersL performance due to concentration quenching, in view of the fact that the trap distribution remained unchanged with varying doping concentration, as manifested by the invariant profile of the thermoluminescence (TL) glow curves (Supplementary Fig. 1c). The corresponding excitation spectrum of the red PersL (i.e., the PersLE spectrum) is given in Fig. 1e, along with the PLE spectrum, the blue radiation of white light emitting diode (W-LED), and the solar spectrum for comparison. The PersLE spectrum displays an absorption maximum at ~380 nm corresponding to the IVCT transition, in contrast with the PLE spectrum where the absorption maximum corresponds to the host excitonic transition (cf. Fig. 1b). This means that the IVCT excitation is more efficient than the host excitonic excitation in generating the red PersL of Pr^3+^, which is most probably due to the presence of carrier trapping and recombination processes in PersL, as will be discussed later. Additional PersLE peaks were also observed corresponding to Pr^3+ 3^H_4_ → ^3^P_J_ transitions. Since they are partially overlapped with the blue radiation spectrum of W-LED, this observation implies that W-LED can be used as a charging source for the red PersL. The sunlight can also act as a charging source in view of the overlap of its spectrum with the PersLE spectra. Finally, owing to the presence of the heavy-ion Gd^3+^, the NGT displays intense X-ray response that is comparable to the X-ray charged PersL material NaYF_4_ (Supplementary Fig. 1d)^27–29^. Therefore, red PersL of NGT:Pr^3+^ can be effectively generated after pre-irradiation with multiple sources ranging from X-ray to blue light (see the top panel of Fig. 1e or Supplementary Fig. 1e), under the thermal disturbance at RT. In addition to thermal stimulation at RT, red luminescence of Pr^3+^ can be stimulated by using low-energy light after ceasing the charging irradiation (i.e., PSL). Figure 1f shows evidently that the faded TSL is improved by photo-stimulation using a 980 nm NIR laser. It means that the charge carries captured at deep traps can be more effectively released by the NIR photo-stimulation than by thermo-stimulation at RT.

Except for the thermo- and NIR photo-stimulation, mechanical stimuli are also able to trigger the release of the stored energy as light emission (i.e., ML). NGT:0.1%Pr^3+^, 0.3%Er^3+^ powders were selected as an example and further encapsulated in optical transparency polydimethylsiloxane (PDMS) elastomers to facilitate the analysis of ML. Figure 1g shows the detailed dimensions of the as-fabricated PDMS composite film that was characterized by SEM from surface, edge and cross-section views. The cross-section SEM images clearly indicate that the thickness of the composite elastomer was ca. 1 mm with NGT:0.1%Pr^3+^, 0.3%Er^3+^ particles tightly embedded in the PDMS matrix. The upper surface is neat while the film edge is slightly rough. Although only a few particles were exposed on the surface to be rubbed, intense red ML (Fig. 1h and Supplementary Fig. 1f) could be observed from the pre-irradiated composite film upon rubbing because of the efficient stress transfer ability of PDMS elastomer. Since friction generates heat, the temperature curve in the process of repeated friction was also measured (Fig. 1h). No significant increase of temperature was detected, implying minor contribution of TSL to the overall luminescence during friction.

### Manipulating emission color in space and time dimensions

**Fig. 2:**
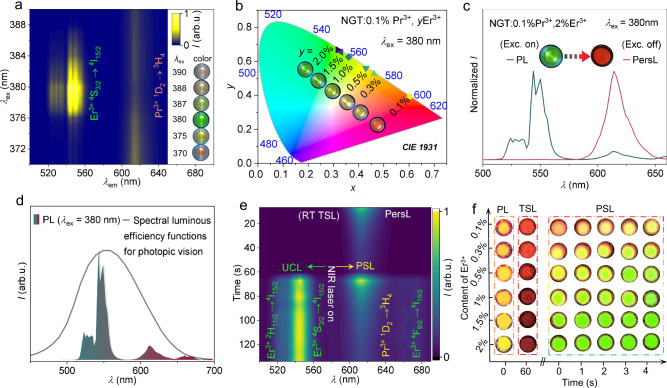
Manipulating emission color in space and time dimensions. **a** Contour plot of the excitation-dependent PL of the NGT:0.1%Pr^3+^, 1.5%Er^3+^. The right sides show photographs of the sample under selected excitations. **b** CIE chromaticity coordinates and corresponding photographs of NGT:0.1%Pr^3+^, *y*Er^3+^ (*y* = 0.1–2%) when fixing the excitation wavelength at 380 nm. **c** Comparison of normalized PL and PersL spectra and the corresponding photographs of NGT:0.1%Pr^3+^, 2%Er^3+^ under/after 380 nm excitation. **d** Spectral sensitivity function of the average human eye under photopic vision^27^ compared with the emission spectrum of NGT:0.1%Pr^3+^, 1.5%Er^3+^ excited by 380 nm. Exc., excitation. **e** The PersL (thermo- stimulated luminescence (TSL) at RT), PSL and up-converted photoluminescence (UCL) spectra of NGT:0.1%Pr^3+^, 0.5%Er^3+^ recorded with time evolution and stimulation. After turning off the 365 nm excitation, the sample shows red PersL at RT. During the PersL decays in a natural way, a 0.2 W 980 nm near-infrared (NIR) CW laser was introduced for photo-stimulation and thus PSL and UCL were generated at the same time along with the PersL. **f** Photographs of NGT:0.1%Pr^3+^, *y*%Er^3+^ (*y* = 0.1–2%) showing manipulation of the overall emission color statically and dynamically via rationally combining PL, TSL and PSL modes. The photographs were, respectively, captured by a smartphone under/after 365 nm irradiation and 0.03 W 980 nm laser irradiation. The laser spot area is ca. 0.25 cm^2^, and the power density is ca. 0.12 W/cm^2^. Source data are provided as a Source Data file.

The partially overlapped excitation bands of Pr^3+^ and Er^3+^ (Supplementary Fig. 1g) implies that the red emission of Pr^3+^ and the green emission of Er^3+^ can be multiplexed to achieve the overall emission color tuning, by controlling the excitation wavelength and/or relative doping concentrations for the doubly doped sample. As shown in Fig. 2a and Supplementary Fig. 2a, by gradually changing the excitation wavelength from 370 to 390 nm, it can switch the emission of NGT:0.1%Pr^3+^, 1.5%Er^3+^ among three colors (red, yellow, and green). On the other hand, fixing the excitation wavelength at 380 nm, the emission color of NGT:0.1%Pr^3+^, *y*Er^3+^ is tunable from red to yellow and then to green when the *y* value increases from 0.1 to 2% (Fig. 2b and Supplementary Fig. 2b). Thus, precise control of excitation wavelength and the dopant ratios enable the manipulation of the overall emission color.

After turning off the excitation, the Pr^3+^, Er^3+^ co-doped sample did not show PersL of the Er^3+^ green emission, but only PersL of the Pr^3+^ red emission (Supplementary Fig. 2c). This provides a luminescence anti-counterfeiting strategy by manipulating the overall emission color temporally. For example, the PL emission color of NGT:0.1%Pr^3+^, 2%Er^3+^ is green under UV light excitation, but it becomes red immediately after removing the excitation (Fig. 2c), exhibiting a high contrast of the overall emission color in the time dimension, which is beneficial to authentication and provides more channels for freewill encoding. Note that the magnitude of the color contrast depends on the relative concentrations of Pr^3+^ and Er^3+^. As the relative content of Er^3+^ increases, the PL emission color shifted from red to green but the red PersL intensity weakened and the time duration shortened (Supplementary Fig. 2c) as a result of the reduced charging absorption of Pr^3+^ along with the enhanced light absorption of Er^3+^ (Supplementary Fig. 2d). We found that NGT:0.1%Pr^3+^, *y*Er^3+^ (*y* ≥ 1.5%) samples present to be good combinations with enhanced color contrast for visual recognition in photopic vision conditions (Fig. 2d)^30^.

Similar to Pr^3+^ singly doped sample, the decay of the red PersL of NGT:Pr^3+^, Er^3+^ can be strengthened by using 980 nm laser photo-stimulation to release the deeply trapped charge carriers (Fig. 2e). However, here the difference is that Er^3+^ also responds to the 980 nm excitation by displaying UCL. As shown in Supplementary Fig. 2e, the emissions at 533, 545, and 660 nm originate from ^2^H_11/2_, ^4^S_3/2_, ^4^F_9/2_ → ^4^I_15/2_ transitions of Er^3+^, respectively. The intensity of each emission is in direct proportion to the *n*^th^ power of the excitation power intensity, where *n* denotes the number of photons involved in the upconversion process. The calculated *n* values for the three emissions are all closed to 2 (Supplementary Fig. 2e), indicating two-photon absorption processes involved in the UCL. Possible upconversion processes and energy level diagrams under 980 nm excitation are shown in Supplementary Fig. 2f. For instance, the green emitting levels ^2^H_11/2_ and ^4^S_3/2_ were populated through the ^4^I_15/2_ → ^4^I_11/2_ ground state absorption (GSA) and the ^4^I_11/2_ → ^4^F_7/2_ excited state absorption (ESA), followed by successive non-radiative relaxation from ^4^F_7/2_ to ^2^H_11/2_ and then to ^4^S_3/2_. The versatile features of NGT:0.1%Pr^3+^, *y*%Er^3+^ inspired us to manipulate the emission color dynamically via rational integrating PL, TSL and PSL modes. As demonstrated in Fig. 2e, f, the emission color of NGT:0.1%Pr^3+^, *y*%Er^3+^ changed from orange-red/yellow (PL) to red (TSL) after switching off 365 nm irradiation. Accordingly, after PersL decays for 60 s, a 0.03 W 980 nm laser was introduced for photo-stimulation, and the emission color changed dynamically with increasing irradiation time because the PSL and UCL were generated simultaneously along with the TSL.

### Physics and interactions involved in the multimode luminescence

**Fig. 3:**
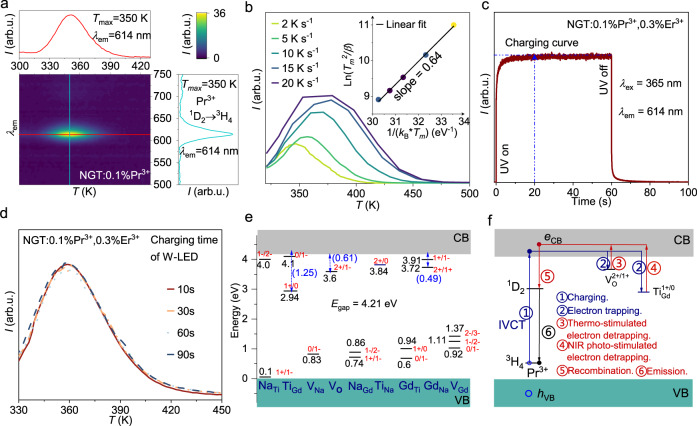
Interaction between defect levels and rare-earth emission centers in NGT. **a** Contour mapping of the TL intensity after 365 nm illumination as a function of emission wavelength and temperature of NGT:0.1%Pr^3+^.The heating rate is 2 K s^−1^. **b** TL glow curves of NGT:0.1%Pr^3+^ at different heating rates *β* from 2 to 20 K s^−1^. The sample was pre-irradiated by a 365 nm UV lamp for 1 min and decayed for 1 min before each measurement. Inset shows the fitting for the determination of the trap depth using the Hoogenstraaten method. **c** Charging curve of NGT:0.1%Pr^3+^, 0.3%Er^3+^ with excitation wavelength *λ*_ex_ = 365 nm. **d** TL glow curves of NGT:0.1%Pr^3+^, 0.3%Er^3+^. The heating rate is 4 K s^−1^. The sample was pre-irradiated by a W-LED (3 W) for different irradiation times (10–90 s). **e** Calculated thermodynamic charge-transition levels of intrinsic point defects. The values in parentheses (in eV) indicate the energy separations of the levels with respect to the host VB maximum (i.e., electron trap depth). *E*_gap_, energy gap. **f** Schematic representation of the mechanistic process for PersL of the red emission of Pr^3+^ in NGT. *e*_CB,_ electrons in CB; *h*_VB_, holes in VB. Source data are provided as a Source Data file.

It is apparent that PersL, PSL, and ML are caused by release of energy stored in traps followed by transfer to Pr^3+^ leading to red emission. As such, we performed TL measurements of NGT:0.1%Pr^3+^ and NGT:0.1%Pr^3+^,0.3%Er^3+^, and the results are shown in Fig. 3a and Supplementary Fig. 3a, respectively. The wavelength-resolved TL spectra for the two samples are essentially the same, showing that Er^3+^ incorporation did not introduce extra intrinsic defects. The TL glow curves span from 300 to 450 K, indicating a continuous distribution of trap depths. The characteristic trap depth *E* (eV) was estimated by using the formula by Hoogenstraaten^31^:

1 $$\frac{\beta E}{{k}_{{{\mbox{B}}}}\cdot {T}_{{{{{{\rm{m}}}}}}}^{2}}={{s}}\exp \left(\frac{-E}{{k}_{{{\mbox{B}}}}\cdot {T}_{{{\mbox{m}}}}}\right)$$

where *β* (K s^−1^) is the heating rate, *k*_B_ is the Boltzmann constant, and *s* (s^−1^) is the frequency factor. As shown in Fig. 3b, the trap depth *E* in NGT:0.1%Pr^3+^, 0.3%Er^3+^ was found to be around 0.64 eV according to the slope of linear fitting of ln (*T*_m_^2^/*β*) against (*k*_B_·*T*_m_)^–1^. This value of trap depth is also within the ideal range of 0.6–0.7 eV suitable for a strong PersL at RT^32^. Figure 3c displays the charging curve of NGT:0.1%Pr^3+^, 0.3%Er^3+^. Under the 365 nm excitation, the intensity increases monotonically and reaches to a stationary value at *t* = 20 s, reflecting the effective charging time of these traps by UV. Figure 3d shows the TL glow curves of NGT:0.1%Pr^3+^, 0.3%Er^3+^ charging by W-LED for different times, which reveal that the effective charging time of these traps by W-LED is 10 s. Undoubtedly, the quick charging speeds guarantee the advantage of convenient authentication in the practical anti-counterfeiting applications.

Since defects contribute significantly to the multimode luminescence^33^, it is essential to understand the interaction between defect levels and rare-earth emission centers in this disordered structure. Accordingly, we have investigated possible intrinsic defects in NGT by using the 2 × 2 × 2 supercell model constructed based on the most probable Na/Gd configuration (see computational details). These defects include four vacancies (V_Na_, V_Gd_, V_Ti_, and V_O_) and six antisites (Na_Gd_, Gd_Na_, Na_Ti_, Ti_Na_, Gd_Ti_, and Ti_Gd_). Hybrid DFT with the HSE06 functional was employed for defect calculations, in view of the fact that the calculated minimum band gap (3.78 eV) among the 838 distinct Na/Gd configurations is close to the experimental value (3.81 eV). Supplementary Figs. 3c, d depicts the calculated defect formation energies (Δ*E*_f_) as a function of the Fermi level (*E*_F_). All three limiting cases for the atomic chemical potentials were considered (see computational details). Considering that NGT:Pr^3+^ was synthesized under an oxidizing atmosphere corresponding to *E*_F_ levels slightly lower than the middle of the band gap, we focus on the Δ*E*_f_ results corresponding to the *E*_F_ levels to within 0.2 eV below the middle position, as indicated by the shaded regions in the figure. It shows that the point defects V_O_, V_Na_, Na_Gd_, Gd_Na_, Ti_Na_, Gd_Ti_, and Ti_Gd_ have minimum Δ*E*_f_ values lower than 1.0 eV, which corresponds to a defect concentration of 1.43 × 10^−3^ as estimated by exp(*−*Δ*E*_f_*/k*_B_*T*) at a synthesis temperature of *T* = 1773 K.

Figure 3e depicts the thermodynamic charge-transition levels of the point defects derived from the formation energies. These levels can be viewed as the energy levels of the defects at different charge states, and their positions relative to the host VB/CB maximum are independent of the atomic chemical potentials. It shows that the oxygen vacancy *V*_O_ provides electron trapping levels *ε*(2+/1+) with depths 0.61 eV, which is appropriate for realizing efficient thermally stimulated PersL at RT. On the other hand, the anti-site Ti_Gd_ introduces electron trapping level ε (1+/0) with depth 1.25 eV, for which the electron captured therein can be released by NIR photo-stimulation at 980 nm.

On the basis of the computational results for intrinsic defects and the experimental PersLE spectral analysis, the dominant mechanism for PersL at RT can be elucidated, as schematically presented in Fig. 3f. Under IVCT charging excitation (arrow 1), electrons are excited directly from the ^3^H_4_ ground state of Pr^3+^ to the host CB, leaving Pr^4+^ behind. The liberated electrons (*e*_CB_) are mobile in the CB and are captured on intrinsic defects V_O_ and Ti_Gd_ with increasing trap depths (arrow 2). After ceasing the charging, the electrons captured at V_O_ will be thermally stimulated into the CB at RT (arrow 3), while the electrons captured at Ti_Gd_ can only be promoted into the CB by NIR light stimulation (arrow 4). The liberated electrons will recombine with Pr^4+^ to produce the Pr^3+^ (^1^D_2_) excited state (arrow 5), which will finally return to the ground state by emitting a photon at ~614 nm (arrow 6). It is noted that, besides the above IVCT charging excitation, the host band-to-band and Pr^3+ 3^H_4_ → ^3^P_J_ charging excitations can also lead to PersL at RT, although with lower efficiency. The PersL mechanisms for these two situations are different from the above dominant mechanism only in the mechanistic detail of electron liberation into the CB. For the host band-to-band charging excitation, *e*_CB_ + *h*_VB_ + Pr^3+^ → *e*_CB_ + Pr^4+^ (*h*_VB_ represents a hole in VB), while for ^3^H_4_ → ^3^P_J_ charging excitation, Pr^3+^ (^3^P_J_) is thermally ionized into *e*_CB_ + Pr^4+^.

### High-level anti­counterfeiting applications

**Fig. 4:**
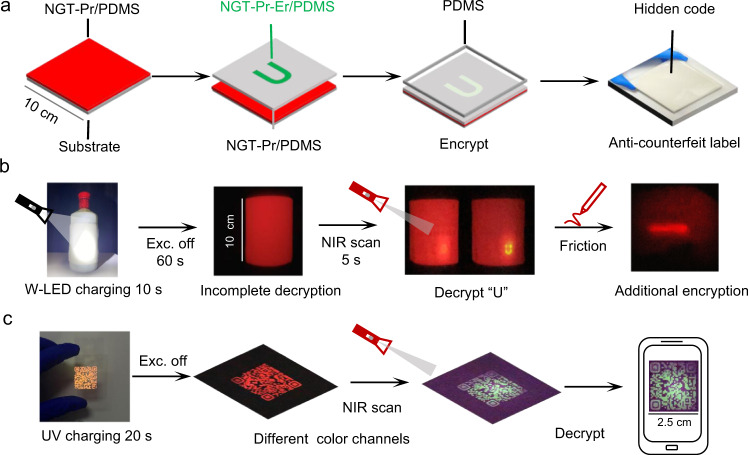
Multilevel anti-counterfeiting application based on real time color-conversion in Pr^3+^, Er^3+^-doped NGT. **a** General design of the hidden two-dimension patterns made with NGT:Pr^3+^, Er^3+^ of different composition. The pattern consists of three layers, which deposited in turn on a glass substrate (10 × 10 cm). The as-prepared multimode luminescent particles and PDMS were mixed and used for different functions: (from left to right) NGT:0.1%Pr^3+^@PDMS as background layer, NGT:0.1%Pr^3+^, 0.1%Er^3+^ @ PDMS as the information layer, transparent PDMS as levelling surface. The anti-counterfeit label with hidden information “U” (1.5 × 0.8 cm) was finally fabricated. **b** The as-prepared flexible anti-counterfeit label is applied to a bottle. Emission profiles of the patterns recorded under different irradiation or stimuli conditions. The power density of the laser used in NIR scan is ca. 0.82 W/cm^2^. **c** Anti-counterfeiting barcode fabricated via screen printing. Barcode (made with NGT: 0.1%Pr^3+^, 0.5%Er^3+^@PDMS) photographs under and after 365 nm UV excitation as well as 980 nm NIR laser photo-stimulation, showing various colors constructing different coding channels. All photos were recorded by a smartphone.

Inspired by the spatio-temporal regulation of the emission color of NGT:*x*Pr^3+^,*y*Er^3+^, we designed a versatile anti­counterfeiting scheme with multichannel encoding, time-resolution display, and multi-stimulus-responsive capabilities. As shown in Fig. 4a and Supplementary Fig. 4, the as-prepared anti-counterfeit label is smooth-faced and the hidden information cannot be observed on the smooth film in any visual angle even under the irradiation of UV/W-LED. Figure 4b shows the decryption process. After switching off the W-LED irradiation the label exhibited significant duration of the red PersL without exposing the hidden information, i.e., it is still incomplete decryption. At this time, an additional 980 nm NIR laser continuously scanning the pre-irradiated film can decrypt the real information “U”. During the scanning, the emission color changes from red to yellow and then to green. Thanks to the tribo-ML and TSL (Supplementary Fig. 5) features of NGT: *x*Pr^3+^, *y*Er^3+^, the almost invisibility PersL can be aroused again by friction or heating, which provide additional channels for encryption. The multicolor emissions, multi-stimuli responsive properties of NGT:Pr^3+^,Er^3+^ also afford opportunities to design photonic barcodes for information encryption. As shown in Fig. 4c and Supplementary Fig. 6a, a series of photonic barcodes with tunable emission colors were prepared, showing three kinds of colors under UV light irradiation as a result of the different ratios of Pr^3+^/Er^3+^ ions. If the luminescence colors store the information, these photonic barcodes will display a higher information capacity. Here, the luminescence colors of these irradiated photonic barcodes can be changed with time duration. After UV irradiation, the emission colors of these barcodes change from red/yellow/green to red and continue to glow for several minutes. We define the longest PersL lifetime as the time when the barcode presents high resolution that can be clearly scanned by a smartphone. For example, the PersL lifetime of NGT:0.1%Pr^3+^, 0.5%Er^3+^ based barcode is 90 s, as shown in Supplementary Fig. 6b. Meanwhile, the color can be changed by 980 nm NIR photo-stimulation (from red/yellow to green), which are ideally suited for real time information encryption.

In summary, we present a design principle for the creation of integrated colorful and dynamic luminescence with distinct emissions and timescales based on a single platform via rare-earth phosphors. We demonstrate versatile defects play the active role in regulating the multimode luminescence in the Pr^3+^ and Er^3+^-doped NaGdTi_2_O_6_ with the disordered site occupation. NaGdTi_2_O_6_:Pr^3+^,Er^3+^ demonstrates a wide spectral charging range (from X-ray to sunlight) and responses to photo-, thermo-, mechano-stimuli, and the emission colors can be manipulated statically and dynamically. Through the combined experimental and theoretical approaches, the Pr^3+^ ↔ Pr^4+^ valence change, oxygen vacancies, and anti-site Ti_Gd_ defects were deemed to be dominating roles in multimode luminescence. The present result provides a theoretical basis for understanding experimental observations and rational designing strategies to tailor multimode luminescence properties. An inexpensive, convenient for authentication, and nondestructive scheme for advanced security and encoding encryption applications were finally demonstrated. Our investigations may enable great feasibility in exploring advanced multimode luminescent materials for broad applications.

## Methods

### Synthesis

NaGd_1-*x*-*y*_Ti_2_O_6_: *x*Pr^3+^, *y*Er^3+^ (*x* = 0.05%, 0.1%, 0.2%, 0.3%, 0.4%, 0.5%, 2%; *y* = 0.1%, 0.5%, 1%, 1.5%, 2%) powders were prepared by a solid-state method. Na_2_CO_3_ (99.9%), TiO_2_ (99.9%), Gd_2_O_3_ (99.99%), Pr_6_O_11_ (99.99%), and Er_2_O_3_ (99.99%) were used as the raw materials. First, stoichiometric amounts of the starting materials were mixed and thoroughly ground in an agate mortar by adding an appropriate amount of ethanol as a dispersing agent. Then, the mixture was transferred to an alumina crucible, and sintered at 1500 ˚C for 4 h in air. After cooling to RT, the samples were ground again for later use.

### Preparation of composite films

Polydimethylsiloxane (PDMS, SYLGARD® 184, Dow Corning) was chosen as the elastic matrix to provide interior stress for NGT:*x*Pr^3+^, *y*Er^3+^. First, 4.0 g of PDMS base resin and 0.4 g of curing agent were mixed in a beaker. Then, 2 g of screened particles (200 mesh) were dispersed in the above PDMS precursor with stirring for 5 min. After curing at 120 ˚C for 10 min, the flexible composites were obtained.

### Preparation of anti-counterfeit labels

The background layer NGT:0.1%Pr^3+^ @PDMS were firstly deposited and solidified on the glass substrate. Then the information layer NGT:0.1%Pr^3+^, 0.1%Er^3+^ @ PDMS were is deposited on the background layer through the mask and then solidified. After that, the mask is removed and the empty part is filled by NGT:0.1%Pr^3+^ @PDMS. Finally, a thin layer of transparent PDMS is covered and solidified on the surface to ensure that the security label is integrated.

### Preparation of barcodes

The barcode was fabricated via screen printing. The as-prepared phosphor was mixed with PDMS and forming into precursor pastes. The pastes pass through the prefabricated barcode pattern and is deposited on the substrate. After heating, the pastes solidify to form the required barcode.

### Characterization

The powder X-ray diffraction (XRD) patterns were collected by an Aeris X-ray diffractometer (PANalytical Corp.) operating at 40 kV and 15 mA with monochromatized Cu Kα radiation (*λ* = 1.5406 Å) and a linear VANTEC detector. The microstructure of as-prepared materials was examined by scanning electron microscope images (SEM) and energy-dispersive X-ray spectroscopy (EDS) (Bruker Nano GmbH Berlin, Germany). PL spectra, PLE spectra, PersL spectra, and decay curves were measured using a high-resolution spectrofluorometer (FLS1000, Edinburgh Instruments, UK) equipped with a Xe lamp as an excitation source and R928 photomultiplier (200 – 900 nm) as a detector. For the PersLE measurements, monochromatic light emitted by a xenon lamp with different wavelength between 250 nm and 550 nm (10 nm per step) were used to excite NGT:0.1%Pr^3+^. After charging for 10 s, the PersL decay curves were recorded and the intensity of PersL at 10 s decay were used as the reference point to plot the PersLE spectrum. To ensure the accuracy of the results, the sample was heated to 550 K to clean the charged energy traps in each measurement. X-ray source (Amptek Mini-X tube with an Mo target and 12 W maximum power output) was used for the X-ray excited PersL spectra and decay curves measurements.

The UCL and PSL spectra were recorded by FLS1000 equipped with an external power tunable 980 nm laser diode array as excitation source. TL glow curves measurements were performed with a TL dosimeter (FJ-427A1) from RT to 500 K. The sample was pre-irradiated at RT, and then it was left for 1 min before the TL measurement. Moreover, an automated TL/optically stimulated luminescence reader (Risφ TL/OSL Da-20, DTU Nutech, Denmark) coupled with a spectrometer (350–1000 nm) (QE65 Pro, Ocean Optics) was used for 3D TL two-dimensional plot measurements. Before measurements, the sample was pre-irradiated at RT. Temperature curve was recorded by a thermal infrared camera. The ML spectra were recorded using a photon-recording system consisting of a fiber optical spectrometer (QE65pro) and a computer. Different light sources were employed to charge the samples, including X-ray tube (10W), UV LED (365 nm, 5W), W-LED (3W) and sunlight. The optical photographs were captured using a smartphone or a Canon 80 D camera.

### Computational method

The crystal defects associated with the trap depths could be revealed by using first-principles calculations. To proceed, however, the occupation disorder of Na/Gd at the 4c site of NGT needs to be properly modeled first. This was carried out by considering all possible configurations of 8Na + 8Gd over the 16 sites of a 2 × 1 × 2 NGT supercell, giving (16)!/(8! × 8!) = 12,870 different configurations to explore short-range order in the Na/Gd distribution. After taking into account the crystal symmetry^34^, the number of crystallographically distinct configurations reduces to 838, which are necessary for density functional theory (DFT) calculations. The relative occurrence probabilities of these configurations (*P*) were then evaluated by:

2 $$P=\frac{1}{Z}\Omega {{{{{\rm{exp }}}}}}\left(-\frac{E}{{k}_{{{\mbox{B}}}}\cdot T}\right)$$

where *Z* is the partition function, Ω is the configuration multiplicity, *E* is the DFT total energy, *k*_B_ is the Boltzmann constant, and *T* (=1773 K) is the synthesis temperature of the material. The results show that the three largest probabilities are 0.255, 0.087, and 0.052, and the other probabilities are each lower than 0.050. In the following, the Na/Gd configuration with the largest occurrence probability was employed for defect calculations.

The atomic structures of defective supercells were fully optimized by minimizing the total energy and Hellmann-Feynman forces to convergence of 10^−6^ eV and 0.01 eV Å^−1^, respectively. Pure DFT-PBE^35^ computations were conducted to investigate the relative occurrence probabilities of the 838 Na/Gd configurations, and hybrid DFT-HSE06^36,37^ computations were performed to calculate the formation energies of point defects, as implemented in the VASP package^38,39^. The Na(3s^1^), Gd(5p^6^6s^2^5d^1^), Ti(4s^2^3d^2^), and O(2s^2^2p^4^) were treated as valence electrons, and their interactions with the respective cores were described by the projected augmented wave (PAW) method^40^. Owing to the large size of the systems and the high computational cost of hybrid DFT, one *k*-point Г was used to sample the Brillouin zone, and the cutoff energy for the plane wave basis was set to 420 eV.

The formation energy of a defect in charge state *q* was calculated using a standard formalism^41^,

3 $${\triangle E}_{{{{{{\rm{f}}}}}}}={E}_{{{{{{\rm{tot}}}}}}}\left({{{{{\rm{defective}}}}}}\right)-{E}_{{{{{{\rm{tot}}}}}}}\left({{{{{\rm{perfect}}}}}}\right)+\mathop{\sum }\limits_{{{{{{\rm{A}}}}}}}{\triangle n}_{A}{\mu }_{A}+q\left({{{{{{\rm{\varepsilon }}}}}}}_{{{{{{\rm{VBM}}}}}}}+{E}_{{{{{{\rm{F}}}}}}}\right).$$

where *E*_tot_ is the total energy of the defective or perfect supercell. Δ*n*_A_ is the number of the species A removed from the perfect supercell, and *μ*_A_ is the corresponding atomic chemical potential. *E*_F_ is the Fermi-level measured from the valence-band maximum (*ε*_VBM_), which was aligned with that of the perfect supercell by the macroscopic averaging approach^42^. The correction to the total energy of charged supercells due to finite-cell size was taken into account by using the scheme proposed in ref. 43. The defect formation energy depends on the chemical potentials of the constituent atoms. In the thermodynamic equilibrium, the chemical potentials are constrained within the following relation

4 $${\mu }_{{{\mbox{Na}}}}+{\mu }_{{{\mbox{Gd}}}}+{\mu }_{{{\mbox{Ti}}}}+{4\mu }_{{{\mbox{O}}}}={\mu }_{{{\mbox{NaGd}}}{{{\mbox{Ti}}}}_{2}{{{\mbox{O}}}}_{6}}$$

where ${\mu }_{{{\mbox{NaGd}}}{{{\mbox{Ti}}}}_{2}{{{\mbox{O}}}}_{6}}$is the total energy of one formula unit of NGT. Since the materials were synthesized in air (i.e., O-rich atmosphere), ${\mu }_{{{{{{\rm{O}}}}}}}$ was first approximated by half the energy of O_2_ plus the effect of temperature and pressure. The atomic chemical potentials of the other species may be further determined by thermodynamic equilibrium conditions of various secondary phases containing the species. Three limiting cases (case A–C) were considered, and the derived *μ*_A_ values therefrom were used to calculate defect formation energies Δ*E*_f_, which represents the lower/upper limits for a point defect at a given Fermi-level under O-rich condition.

The atomic chemical potentials for the other species were determined by thermodynamic equilibrium conditions of various secondary phases containing the species. Herein, we consider the following three limiting cases (A–C).

Case A (the upper limits for *μ*_Na, Ti_ and the lower limit for *μ*_Gd_):

5 $${\mu }_{{{\mbox{Na}}}}=1/2\left({\mu }_{{{{\mbox{Na}}}}_{2}{{\mbox{O}}}}-{\mu }_{{{\mbox{O}}}}\right)$$

6 $${\mu }_{{{\mbox{Ti}}}}={\mu }_{{{{\mbox{TiO}}}}_{2}}-2{\mu }_{{{\mbox{O}}}}$$

7 $${\mu }_{{{\mbox{Gd}}}}={\mu }_{{{\mbox{NaGd}}}{{{\mbox{Ti}}}}_{2}{{{\mbox{O}}}}_{6}}-{\mu }_{{{\mbox{Na}}}}-{2\mu }_{{{\mbox{Ti}}}}-{6\mu }_{{{\mbox{O}}}}$$

Case B (the upper limits for *μ*_Na, Gd_ and the lower limit for *μ*_Ti_):

8 $${\mu }_{{{\mbox{Na}}}}=1/2\left({\mu }_{{{{\mbox{Na}}}}_{2}{{\mbox{O}}}}-{\mu }_{{{\mbox{O}}}}\right)$$

9 $${\mu }_{{{\mbox{Gd}}}}=1/2\left({\mu }_{{{{\mbox{Gd}}}}_{2}{{{\mbox{O}}}}_{3}}-{3\mu }_{{{\mbox{O}}}}\right)$$

10 $${\mu }_{{{\mbox{Ti}}}}={1/2}(\mu _{{{\mbox{NaGd}}}{{{\mbox{Ti}}}}_{2}{{{\mbox{O}}}}_{6}}-{\mu }_{{{\mbox{Na}}}}-{\mu }_{{{\mbox{Gd}}}}-{6\mu }_{{{\mbox{O}}}})$$

Case C (the upper limits for *μ*_Gd, Ti_ and the lower limit for *μ*_Na_):

11 $${\mu }_{{{\mbox{Gd}}}}=1/2\left({\mu }_{{{{\mbox{Gd}}}}_{2}{{{\mbox{O}}}}_{3}}-{3\mu }_{{{\mbox{O}}}}\right)$$

12 $${\mu }_{{{\mbox{Ti}}}}={\mu }_{{{{\mbox{TiO}}}}_{2}}-2{\mu }_{{{\mbox{O}}}}$$

13 $${\mu }_{{{\mbox{Na}}}}={\mu }_{{{\mbox{NaGd}}}{{{\mbox{Ti}}}}_{2}{{{\mbox{O}}}}_{6}}-{\mu }_{{{\mbox{Gd}}}}-2{\mu }_{{{\mbox{Ti}}}}-{6\mu }_{{{\mbox{O}}}}$$

The total energies of the bulk materials Na_2_O, Gd_2_O_3_, and TiO_2_ were calculated to determine the relevant chemical potentials.

The thermodynamic charge-transition levels (or defect levels) within the band gap correspond to the Fermi-level position at which a transition occurs from one charge state (*q*) to another (*q*′). The level position $\varepsilon (q/q\hbox{'}){{\mbox{}}}$ with reference to the host VBM can be calculated by

14 $$\varepsilon ({q}/{q}{\prime} )=\frac{\triangle {{E}}_{{{\mbox{f}}}}({q}{\prime} {;}{{E}}_{{{\mbox{F}}}}=0)-\triangle {{E}}_{{{\mbox{f}}}}({q;}{{E}}_{{{\mbox{F}}}}=0)}{{q}-{q}{\prime} }$$

where Δ*E*_f_ (*q* or *q*′; *E*_F_ = 0) are the formation energies of the defect in charge state *q* or *q*′ when the Fermi-level is set at 0 eV.

## Supplementary information

Supplementary Information

## Data Availability

The experiment data that support the findings of this study are included in this published article and its supplementary information files and are available from the corresponding author upon request. Source data are provided with this paper.

## Source data

Source Data
