# Supplementary material for: Interplay of defect levels and rare earth emission centers in multimode luminescent phosphors
Source: Nat Commun. 2022 Dec 8;13:7589. doi: 10.1038/s41467-022-35366-3 (PMC9732309; doi:10.1038/s41467-022-35366-3)
Supplement: Supplementary file 1 — Supplementary Information [file 41467_2022_35366_MOESM1_ESM.pdf]

Supplementary Information

Interplay of defect levels and rare earth emission centers in multimode luminescent phosphors

Xinquan Zhou[1], Lixin Ning[2*] Jianwei Qiao[1], Yifei Zhao[1], Puxian Xiong[1] & Zhiguo Xia[1,3*]

[1]State Key Laboratory of Luminescent Materials and Devices, Guangdong Provincial Key Laboratory of Fiber Laser Materials and Applied Techniques, Guangdong Engineering Technology Research and Development Center of Special Optical Fiber Materials and Devices, School of Materials Science and Engineering, South China University of Technology, Guangzhou, 510641, China. [2]Anhui Key Laboratory of Optoelectric Materials Science and Technology, Key Laboratory of Functional Molecular Solids, Ministry of Education, Anhui Normal University, Wuhu, 241000, China. [3]School of Physics and Optoelectronics,South China University of Technology,Guangzhou, 510641, China.

Corresponding Author

ninglx@mail.ahnu.edu.cn (L. Ning), xiazg@scut.edu.cn (Z. Xia)

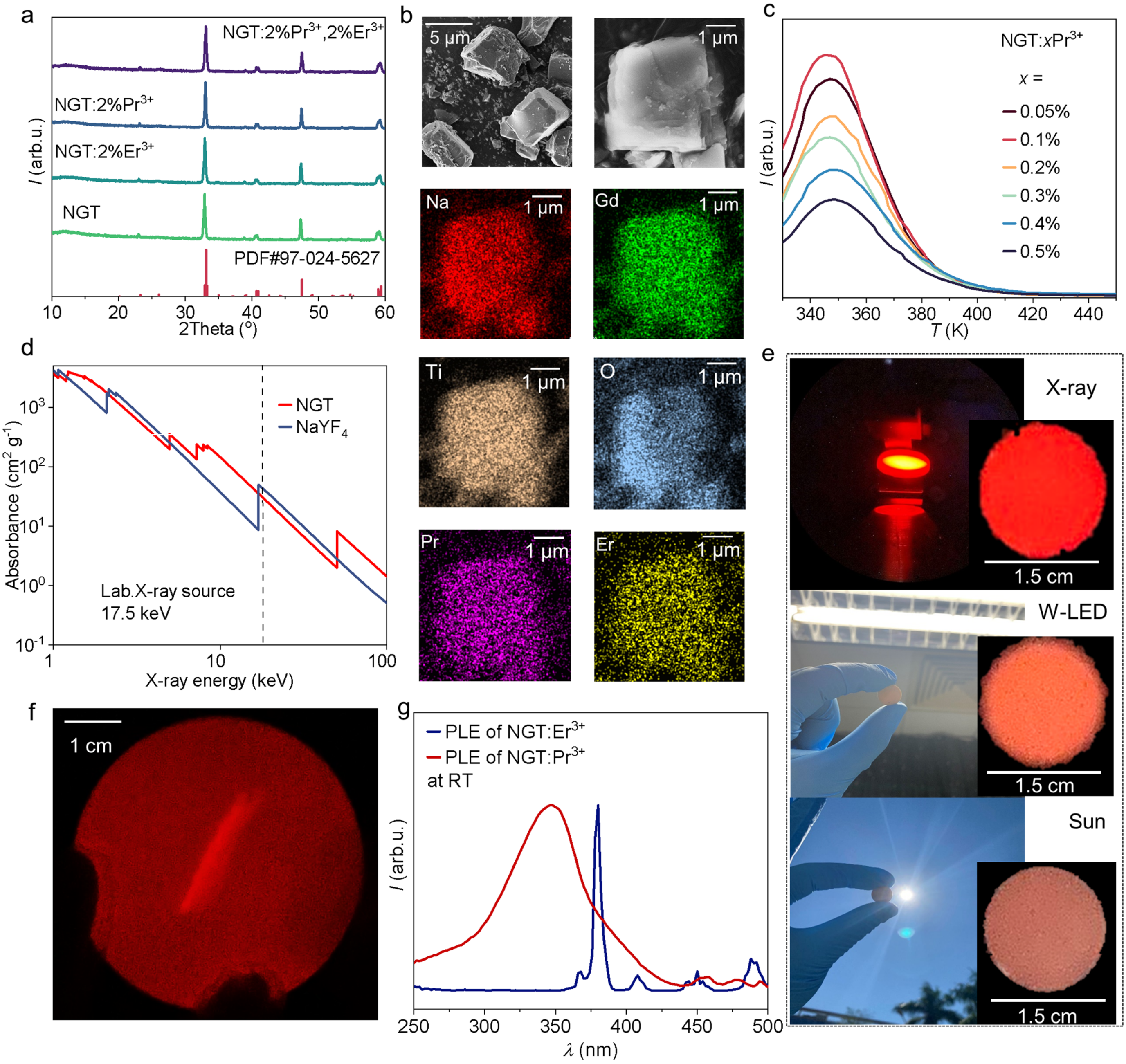

Supplementary Fig. 1 | Structure, morphology and spectra characterizations of NGT:Pr^{3+},Er^{3+}. a X-ray diffraction patterns of NGT, NGT:2%Pr^{3+}, NGT:2%Er^{3+} and NGT:2%Pr^{3+}, 2%Er^{3+}. **b** SEM image of NGT:2%Pr^{3+}, 2%Er^{3+} microcrystal particles and EDS elemental mapping images of selected particles. **c** TL glow curves of NGT: $x$$Pr^{3+}$ (x = 0.05% - 0.5%) after UV irradiation for 30 s. The heating rate is 2 K s^{-1}. **d** Absorption spectra of NGT and $NaYF_4$ as a function of X-ray energy. Attenuation coefficients were obtained from ref.[1]. **e** PL and PersL photographs under/after excited by different light sources (W-LED, sunlight, X-ray). **f** Tribo-ML photograph of the flexible composite film consisting of NGT:0.1%Pr^{3+}, 0.3%Er^{3+} and PDMS. **g** Overlap of RT PLE spectra of Pr^{3+} / Er^{3+} singly doped NGT. Source data are provided as a Source Data file.

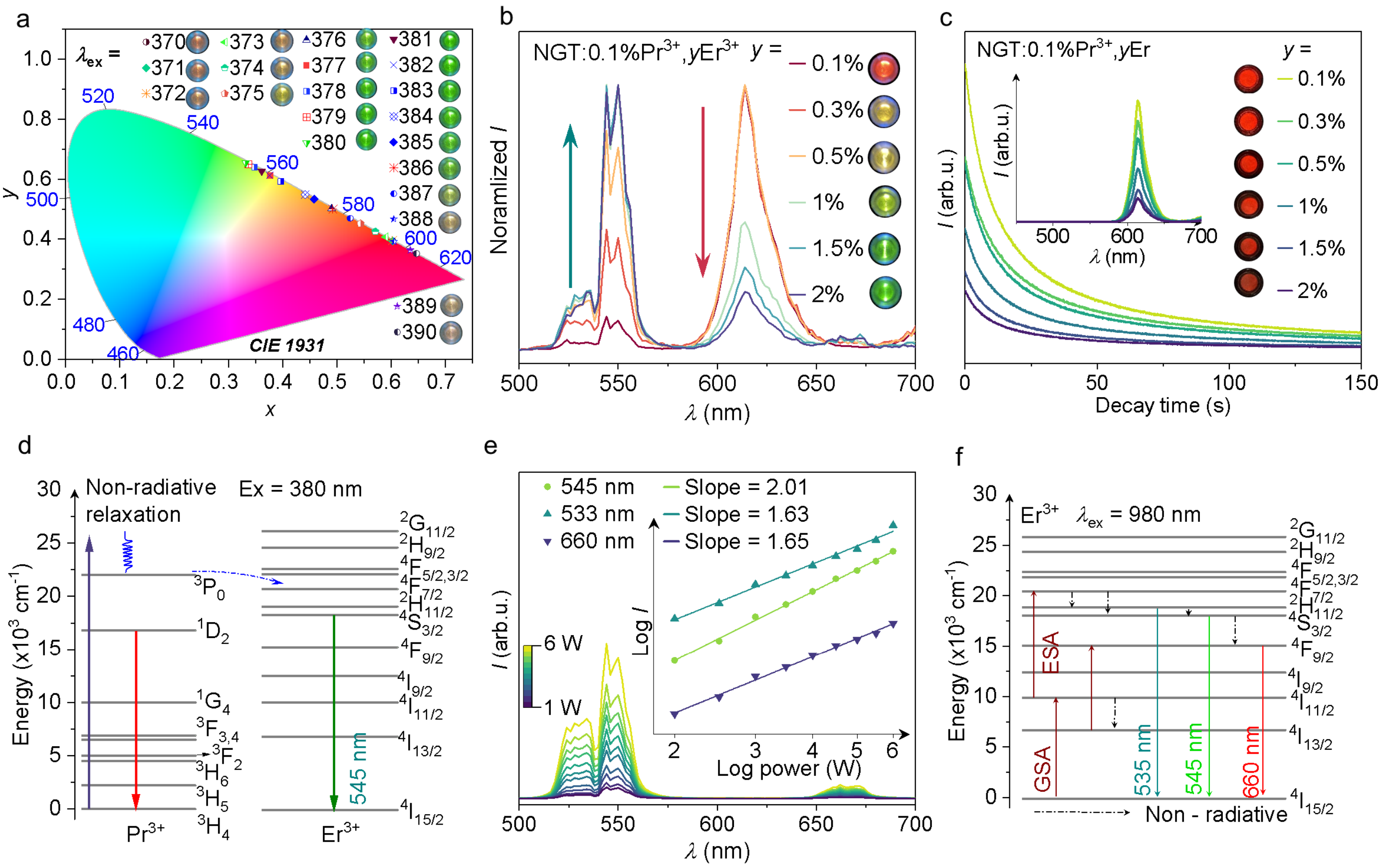

Supplementary Fig. 2 | DCL and UCL characterization of NGT:Pr^{3+},Er^{3+}. a Photographs and CIE chromaticity coordinates of NGT:0.1%Pr^{3+}, 1.5%Er^{3+} under different excitations. **b** PL spectra and photographs of NGT:0.1%Pr^{3+}, *y*Er^{3+} (*y* = 0.1% - 2%) under 380 nm excitation. **c** PersL decay curves of NGT:0.1%Pr^{3+}, *y*Er^{3+} (*y* = 0.1% - 2%). Inset shows their PersL spectra and photographs. All samples were pre-irradiated by a 380 nm for 1 min before each measurement and picture capture. **d** Energy level scheme of Pr^{3+} and Er^{3+} ions, and possible energy transfer process involving $^3P_0$ level of Pr^{3+} and $F_{7/2}$ level of Er^{3+} [2]. **e** Dependence of the UCL spectra on the 980 nm laser powers. Inset shows double logarithmic plots of excitation power versus UCL intensity of Er^{3+} in NGT:0.1%Pr^{3+}, 0.5%Er^{3+}. **f** Energy level diagrams and corresponding possible UC processes. Source data are provided as a Source Data file.

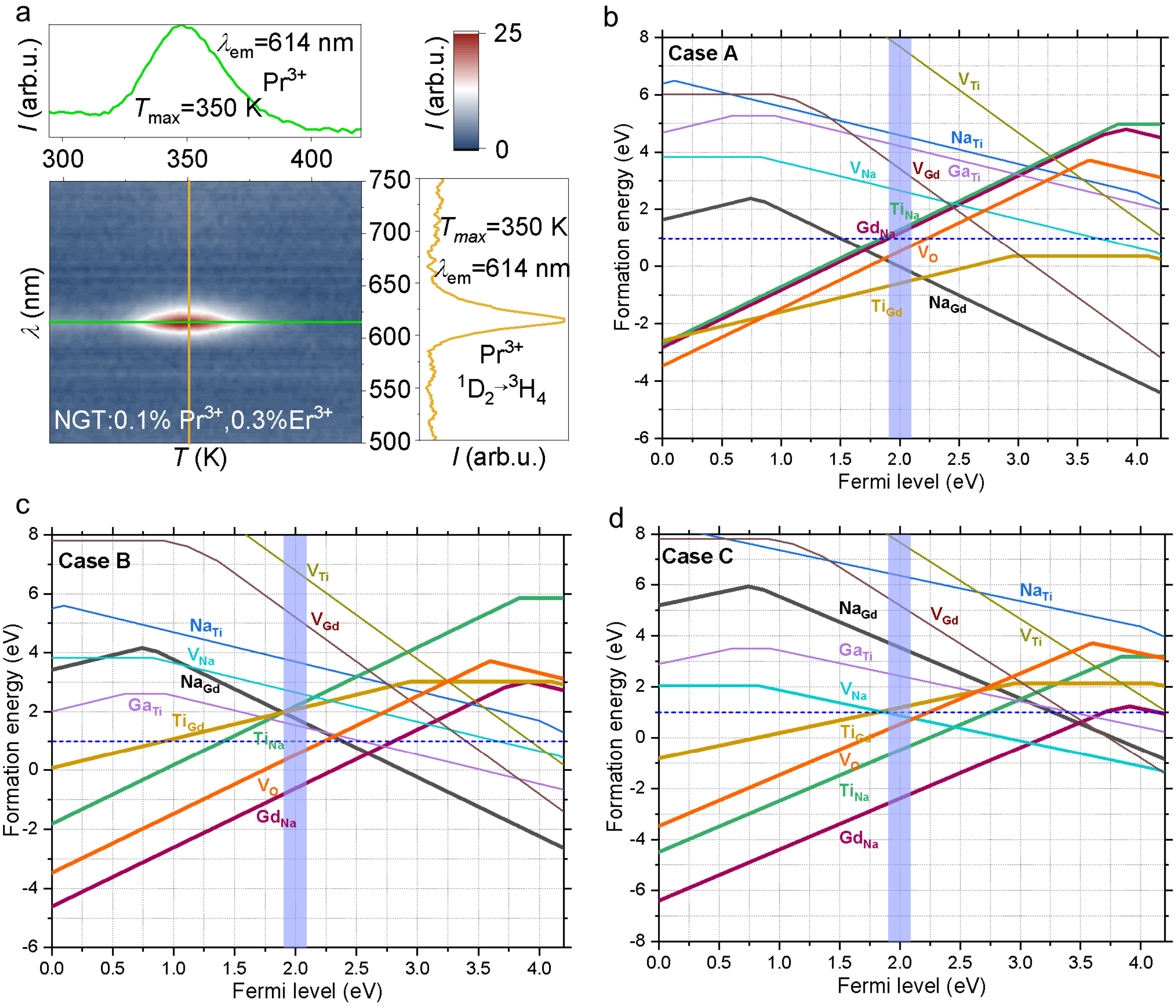

Supplementary Fig. 3 | Formation energies of intrinsic point defects in NGT. a Contour mappings of the TL intensity after 365 nm illumination as a function of emission wavelength and temperature of NGT:0.1%Pr^{3+}, 0.3%Er^{3+}. **b-d** Calculated formation energies of intrinsic point defects in different charge states as a function of the Fermi level position in the band gap of NGT in three limiting cases (A−C) for the atomic chemical potentials. Source data are provided as a Source Data file.

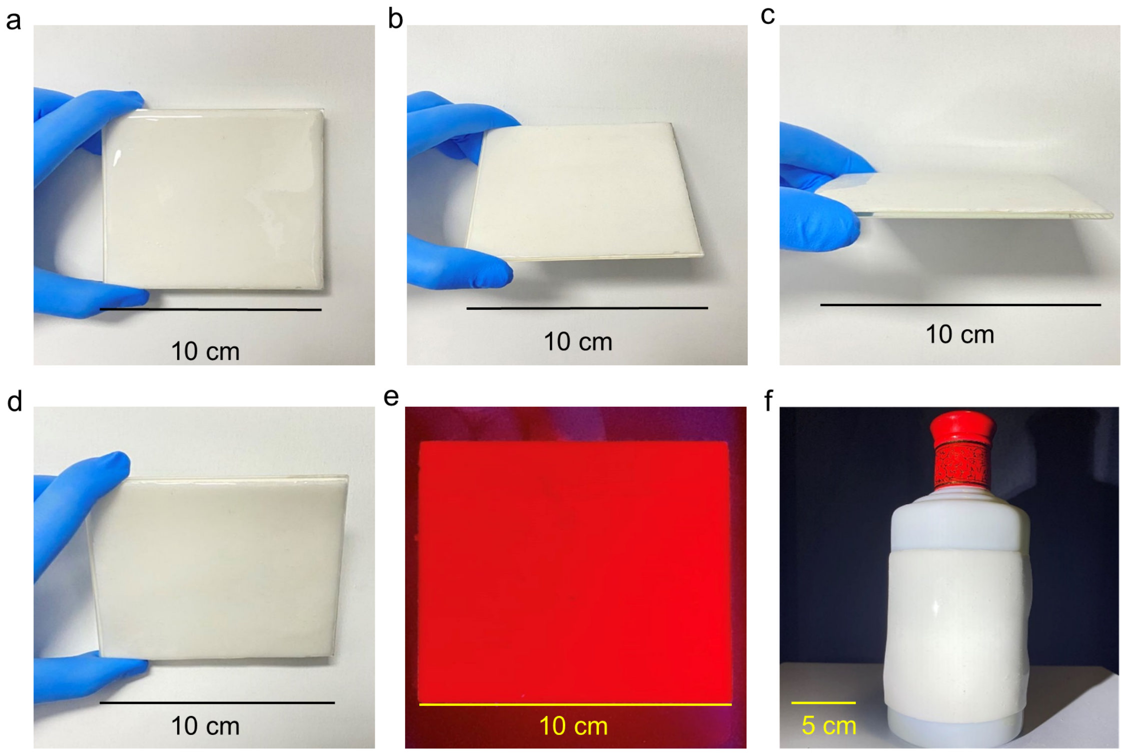

Supplementary Fig. 4 | Photographs of as-prepared films. Observing the as-prepared anti-counterfeiting flexible film from different angles and under different light exposure environment.

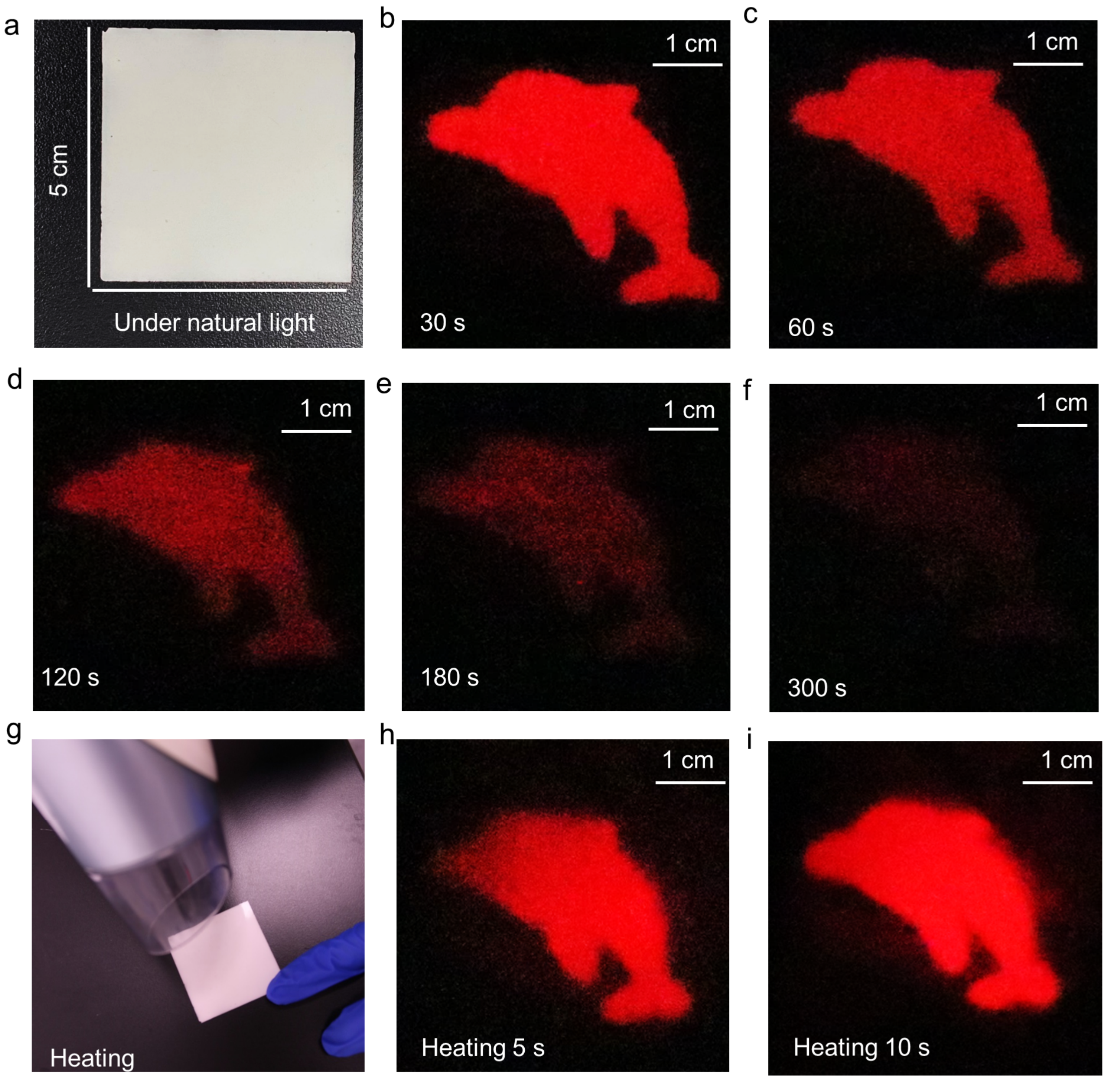

Supplementary Fig. 5 | Demonstration of the anti-counterfeiting process by TSL. a Photograph of the as-prepared anti-counterfeit label made by NGT@PDMS and NGT:0.1%Pr^{3+}, 0.1%Er^{3+} @PDMS under natural light. **b-f** PersL photographs of the label under dark room at various decay times after exposing the label to natural light for 1 min. **g** Photograph of heating process using a common hair dryer as heater. **h-i** Photographs of thermos-stimulated pattern at different heating times.

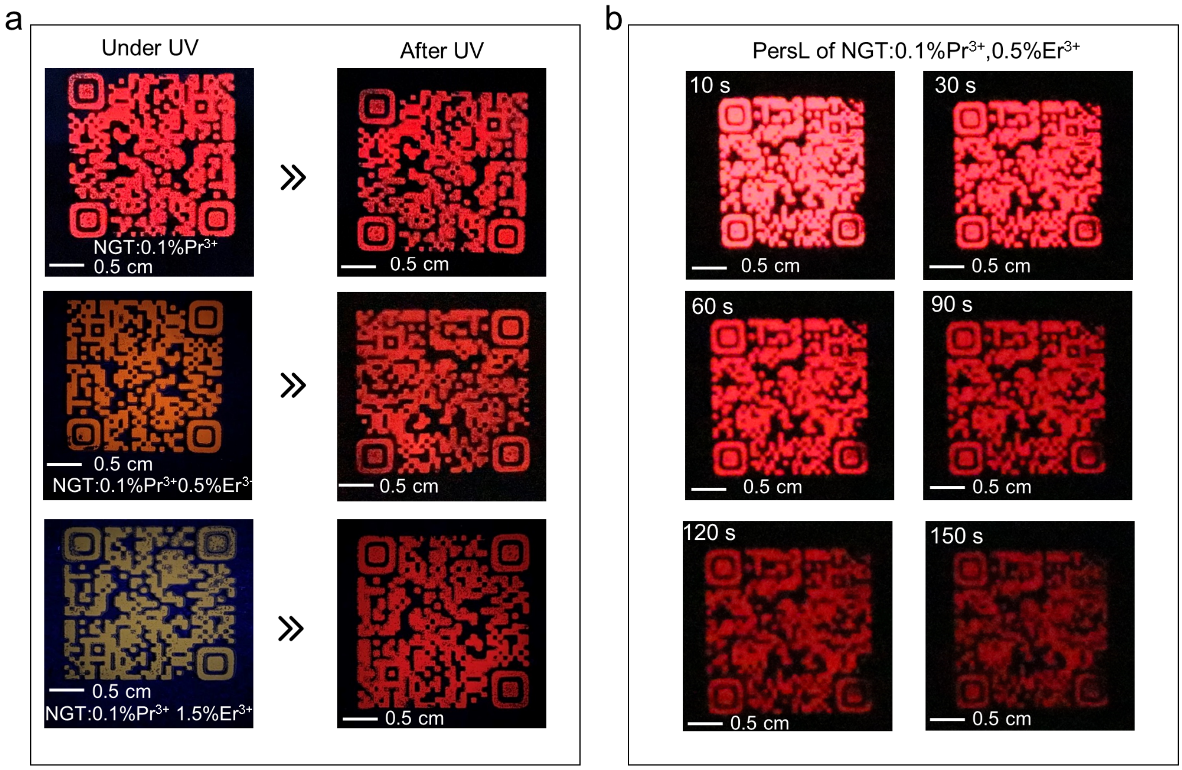

Supplementary Fig. 6 | Emission color evlution and PersL decay of the as-prepared barcodes. a Photographs of NGT:*x*Pr^{3+},*y*Er^{3+} (*x* = 0.1%, *y* = 0 (red), 0.5% (orange), 1.5% (pistac)) barcodes under/after 365 nm UV excitation. These photos were recorded by a smart phone. **b** PersL photographs of the barcode made with NGT: 0.1%Pr^{3+}, 0.5%Er^{3+}@PDMS after 365 nm UV excitation for 30 s.

Supplementary references

1. Berger, M. J. et al., XCOM: Photon cross sections database https://dx.doi.org/10.18434/T48G6X.

2. Zhang, F. et al. Visible luminescence properties of Er^{3+}–Pr^{3+} codoped fluorotellurite glasses. *Opt. Mater.* **41**, 112-115 (2015).
